# Characterization of a newly developed chicken 44K Agilent microarray

**DOI:** 10.1186/1471-2164-9-60

**Published:** 2008-01-31

**Authors:** Xianyao Li, Hsin-I Chiang, James Zhu, Scot E Dowd, Huaijun Zhou

**Affiliations:** 1 Texas Agricultural Experiment Station, Texas A&M University, College Station, TX, USA; 2 USDA Agriculture Research Service Plum Island Animal Disease Center, Orient Point, NY, USA; 3 USDA Agriculture Research Service, Livestock Issues Research Unit, Lubbock, TX, USA

## Abstract

**Background:**

The development of microarray technology has greatly enhanced our ability to evaluate gene expression. In theory, the expression of all genes in a given organism can be monitored simultaneously. Sequencing of the chicken genome has provided the crucial information for the design of a comprehensive chicken transcriptome microarray. A long oligonucleotide microarray has been manually curated and designed by our group and manufactured using Agilent inkjet technology. This provides a flexible and powerful platform with high sensitivity and specificity for gene expression studies.

**Results:**

A chicken 60-mer oligonucleotide microarray consisting of 42,034 features including the entire Marek's disease virus, two avian influenza virus (H5N2 and H5N3), and 150 chicken microRNAs has been designed and tested. In an important validation study, total RNA isolated from four major chicken tissues: cecal tonsil (C), ileum (I), liver (L), and spleen (S) were used for comparative hybridizations. More than 95% of spots had high signal noise ratio (SNR > 10). There were 2886, 2660, 358, 3208, 3355, and 3710 genes differentially expressed between liver and spleen, spleen and cecal tonsil, cecal tonsil and ileum, liver and cecal tonsil, liver and ileum, spleen and ileum (*P *< 10^-7^), respectively. There were a number of tissue-selective genes for cecal tonsil, ileum, liver, and spleen identified (95, 71, 535, and 108, respectively; *P *< 10^-7^). Another highlight of these data revealed that the antimicrobial peptides GAL1, GAL2, GAL6 and GAL7 were highly expressed in the spleen compared to other tissues tested.

**Conclusion:**

A chicken 60-mer oligonucleotide 44K microarray was designed and validated in a comprehensive survey of gene expression in diverse tissues. The results of these tissue expression analyses have demonstrated that this microarray has high specificity and sensitivity, and will be a useful tool for chicken functional genomics. Novel data on the expression of putative tissue specific genes and antimicrobial peptides is highlighted as part of this comprehensive microarray validation study. The information for accessing and ordering this 44K chicken array can be found at

## Background

The chicken, being the first farm animal with a completely sequenced genome, has become an important animal model in the fields of evolution, development, immunology, oncology, cell biology, virology, and genetics [1,2]. Candidate genes, QTL, and molecular markers have been widely utilized to reveal the genetic basis of economically important traits in chickens [3-5]. There are also many new genetic and bioinformatics resources available that are based upon chicken genome information, including genetic and physical maps [6], EST databases [7], and SNP maps [1,8]. Global gene expression profiling will provide a complementary tool improving our ability to study regulation of complex and economically important traits in chickens.

The development of high-throughput microarray has accelerated the study of gene expression by interrogating thousands of genes simultaneously [9-11]. Microarray technologies provide an important tool to infer gene networks and to identify highly conserved genetic pathways in plants and animals. There have been many important studies contributing to gene expression profiling in agricultural animals including pigs [12,13], rabbits [14], and cattle [15,16]. Several chicken cDNA or oligonucleotide probe (oligo) arrays have also been developed and utilized in gene expression studies. These arrays include a 3,011 lymphocyte array [17], a 3,072 intestinal array [18], an 11K heart specific array [19], a 14,718 macrophage specific array [20], a 13K cDNA transcriptome array [21], a 5K immune related array [21,22], a 20K long oligo chicken genome array [23], and a 33K Affymetrix chicken genome array [24].

Short and long oligo arrays have several advantages over cDNA arrays in terms of specificity, sensitivity, and reproducibility [25]. Both microarray technologies can provide comprehensive and reliable data for global expression analyses. However, oligos are more uniform in concentrations and annealing temperature, more gene-specific, flexible, and economic. Long oligos can provide increased signal intensity compared to short ones [26,27]. Long oligo arrays generated by Agilent Technology may be able to detect down to single transcript per cell [25]. This 60-mer 44K chicken whole genome custom array which was developed by our group and manufactured using the Agilent Technology will provide a comprehensive and powerful functional genomics tool for the agricultural community.

## Results

### Genes selected on the array

**Figure 1:**
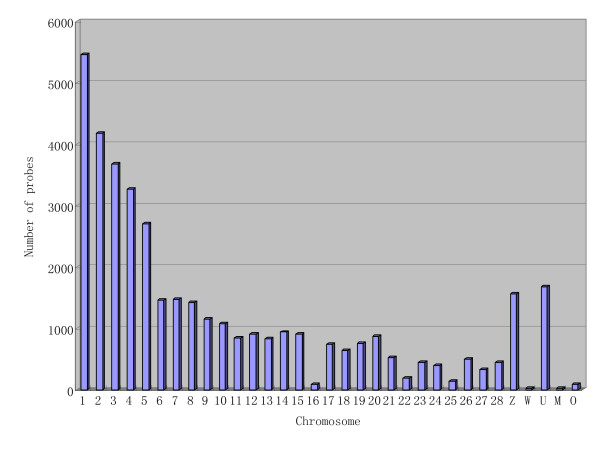
**Number of probes in the microarray represented in each chromosome**. U represents ChrUn_random; M represents mitochondrial sequence; O represents other chromosome, like chrE22, E64.

A total of 42,034 probes were designed based on the whole chicken genome sequence including autosomes, sex chromosomes, unlocalized chromosomes (i.e. E22C19W28, E26C13 and E50C23), and mitochondria (Figure 1), plus 1264 positive control features and 153 negative control features. Chicken chromosomes range from 0.15 Mb to 188.2 Mb [6]. In order to calculate the probe density (number of probes per Mb) on each chromosome, the number of probes targeted to each chromosome was divided by the length of the chromosome. The probe density ranged from 28 probes per Mb (Chr. 16) to 445 probes per Mb (Chr. 2), with a mean value of 76. This array also included probes designed from 150 chicken microRNA, 43 Marek's disease virus genes, and 20 avian influenza virus genes (10 H5N2 and 10 H5N3 genes).

### Array quality

The signal-to-noise ratio (SNR) for each element was calculated using the difference of the median intensity, minus the median background, divided by the standard deviation of the background [28]. The percentage of high quality spots (SNR > 10) were calculated as the number of high quality spots divided by the total number of spots on the array. For all 24 arrays, the average percentage of high quality spots was determined to be 96.55 ± 4.89%.

**Figure 2:**
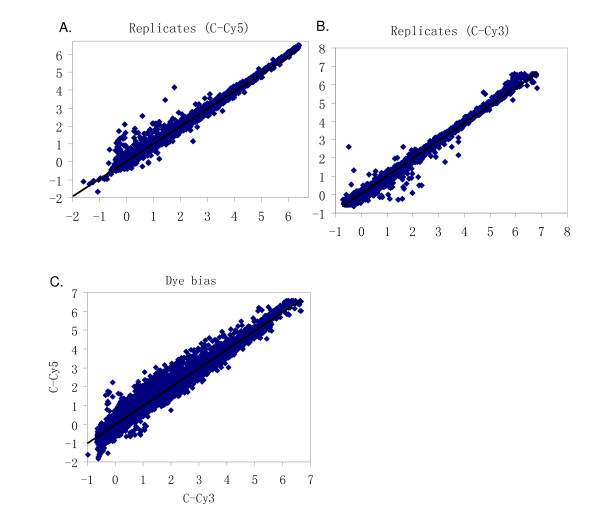
**The correlation of signal intensities between biological replicates and dye swaps**. A. The correlation of signal intensities between two individual cecal tonsil (C) samples labeled with Cy5, Y = 0.98 X+0.0057, R^2 ^= 0.99. B. The correlation of signal intensities between two individual cecal tonsil (C) samples labeled with Cy3, Y = 0.98 X, R^2 ^= 0.99. C. The correlation of signal intensities between the same cecal tonsil (C) sample labeled with Cy3 and Cy5, Y = 0.99 X, R^2 ^= 0.98.

To evaluate the array quality, two comparisons were carried out: (1) two biological replicates from the same tissue labelled with the same dye and (2) the same samples labelled with Cy5 and Cy3. The correlation coefficients of signal intensities between the two biological replicates and between the two different dyes compared among the same samples (dye swap) were calculated by JMP 5.5 (SAS Institute, Cary, NC) (Figure 2). The correlation coefficients between two biological replicates of cecal tonsil labelled with Cy5 or Cy3 were 0.99, 1.00, respectively. The regression lines between two biological replicates of cecal tonsil labelled with Cy5 or Cy3 were y = 0.9779x + 0.0057 (R^2 ^= 0.99) and y = 0.9778x (R^2 ^= 0.99), respectively (Figures 2A, B). Dye swaps were utilized throughout this study in order to avoid the dye bias. The correlation coefficient and regression line between cecal tonsils labelled with Cy5 and Cy3 were 1.00 and y = 0.99x (R^2 ^= 0.98), respectively (Figure 2C).

### Gene expression in different tissues

Before normalization, signal intensities of each feature were filtered against negative controls in the array. The ratio of signal intensity for each gene and the average signal intensity of negative control elements were calculated. An arbitrary ratio of 1.5 was used to determine if a particular gene was expressed in a given tissue. It was found that 43.83% of all genes on the array were expressed within all four tissues. Looking at each tissue individually, it was found that 71.11%, 80.05%, 75.37%, and 80.22% of the genes on the array were expressed in cecal tonsil, ileum, liver, and spleen, respectively.

A comparative study was conducted by comparing gene expression profiles between each of the four selected tissues (cecal tonsil, ileum, liver, and spleen). There were 3710, 3355, 3208, 2886, 2660, and 358 genes significantly and differentially expressed between spleen and ileum, liver and ileum, liver and cecal tonsil, liver and spleen, spleen and cecal tonsil, and cecal tonsil and ileum at the cut-off of *P *< 10^-7^. The corresponding false discovery rate (FDR) for each comparison was calculated and shown to be 4.46 × 10^-7^, 4.14 × 10^-7^, 4.37 × 10^-7^, 5.02 × 10^-7^, 7.39 × 10^-7 ^and 9.11 × 10^-6^, respectively. Out of the 150 chicken microRNAs included in this microarray, it was shown that 15, 36, 31, 24, 15, and 11 microRNAs were differentially expressed when comparing spleen and ileum, liver and ileum, liver and cecal tonsil, liver and spleen, spleen and cecal tonsil, and cecal tonsil and ileum (*P *< 0.05).

**Figure 3:**
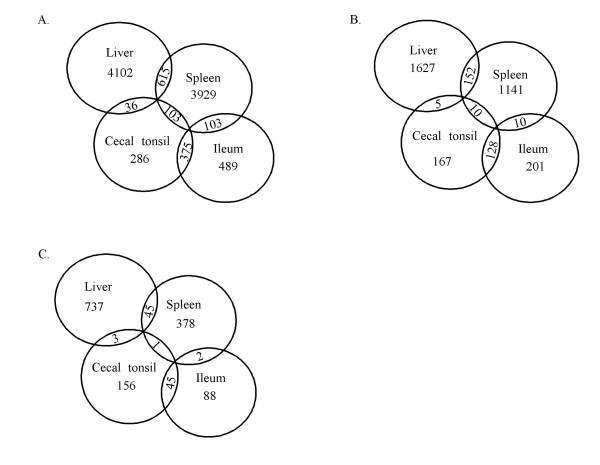
**Venn diagram showing the number of specifically expressed genes in each tissue**. A. Gene number at cut-off *P *values of 10^-3^. B. Gene number at cut-off *P *values of 10^-5^. C. Gene number at cut-off *P *values of 10^-7^.

There were three pairs of tissue gene expression comparisons performed for each tissue as part of this study. These comparisons were used to obtain a list of genes that are specifically expressed in each tissue (Figure 3). In summary, there were 286, 489, 4102, and 3929 genes significantly expressed in cecal tonsil, ileum, liver, and spleen (*P *< 10^-3^), respectively; 167, 201, 1627, and 1141 genes at cut-off *P *value of 10^-5^, and 156, 88, 737, and 378 genes at *P *< 10^-7^.

Fold change is an indication of relative gene expression differences. It is considered that genes, which are expressed at a higher level in one tissue compared to all other tissues, are "tissue-selective" genes [29]. Those genes which are significantly expressed with at least two-fold higher expression in one tissue when compared to the other three tissues are considered to be selectively expressed in this tissue. The data on the selectively expressed genes at three different cut-off *P *values (10^-3^, 10^-5^, and 10^-7^) as determined in this study are listed in Table 1. There were 120, 153, 857, and 541 genes selectively expressed in the cecal tonsil, ileum, liver and spleen at *P *< 10^-3^, respectively. There were 103, 115, 736, and 291 selective genes respectively at a cut-off *P *value of 10^-5 ^and there were 95, 71, 535, and 108 selective genes at a cut-off *P *value of 10^-7^. The selectively expressed genes expressed at *P *< 10^-7 ^are listed in the additional data files 1, 2, 3, 4.

**Table 1:** The number of tissue selective genes at certain cut-off *P *values

Cut-off	Cecal tonsil	Ileum	Liver	Spleen
10^-3^	120	153	857	541
10^-5^	103	115	736	291
10^-7^	95	71	535	108

### Gene ontology

**Figure 4:**
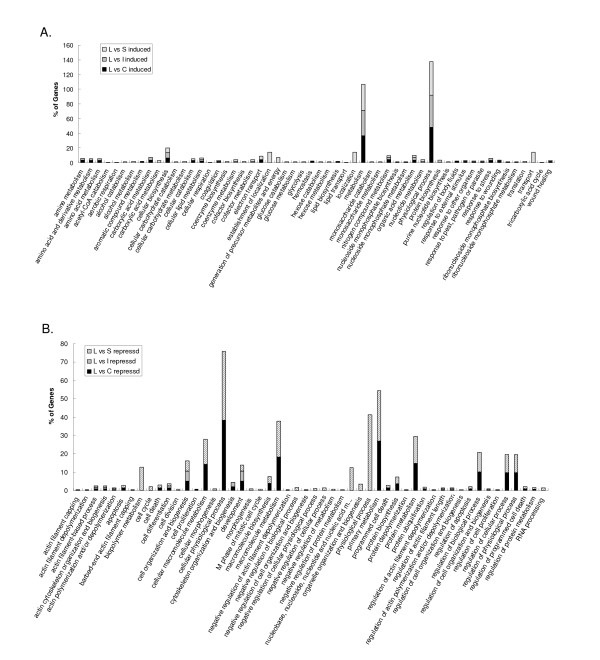
**Significantly enriched Gene Ontology (GO) terms for Biological Process classification of the differentially expressed genes**. A. Up regulated genes between liver and cecal tonsil, liver and ileum, and liver and spleen. B. Down regulated genes between liver and cecal tonsil, liver and ileum, and liver and spleen. nucleobase, nucleoside, nucleotide and nucleic acid ...: nucleobase, nucleoside, nucleotide and nucleic acid metabolism. Percentage shown in Y-axis was calculated as genes in each GO term divided by all up regulated or down regulated genes in each comparison.

**Figure 5:**
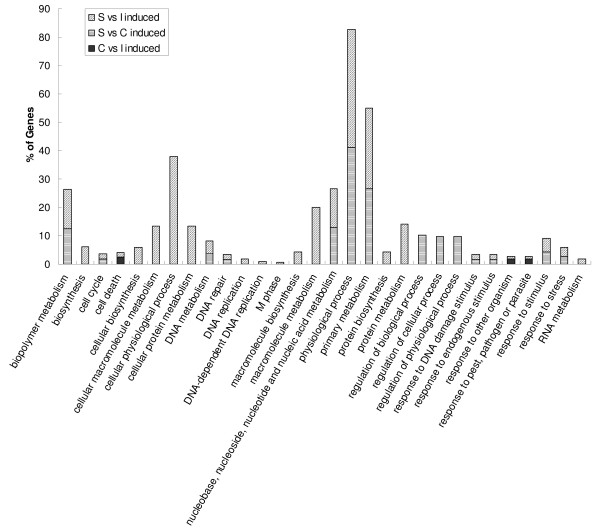
**Significantly enriched Gene Ontology (GO) terms for Biological Process classification of the differentially up regulated genes between cecal tonsil and ileum, spleen and cecal tonsil, spleen and ileum**. Percentage shown in Y-axis was calculated as genes in each GO term divided by all up regulated or down regulated genes in each comparison.

**Figure 6:**
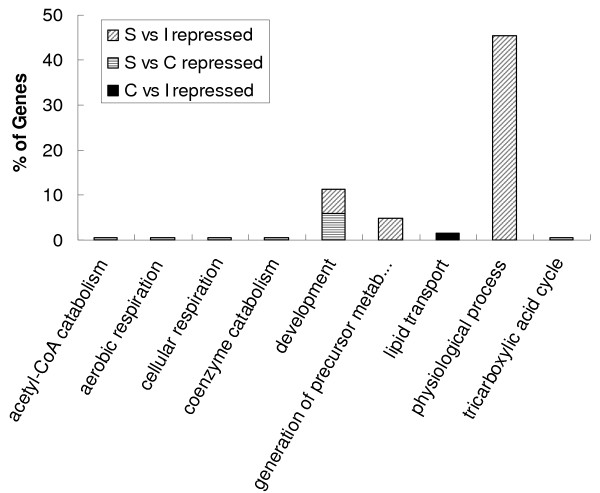
**Significantly enriched Gene Ontology (GO) terms for Biological Process classification of the differentially down regulated genes between cecal tonsil and ileum, spleen and cecal tonsil, spleen and ileum**. Generation of precursor metab...: generation of precursor metabolites and energy. Percentage shown in Y-axis was calculated as genes in each GO term divided by all up regulated or down regulated genes in each comparison.

Functional category enrichment evaluation based on the gene ontology (GO) was performed on the differentially expressed genes for each tissue comparison (Figure 4, 5, 6). There are three components to a GO annotation: cellular component (CC), molecular function (MF), and biological process (BP). Biological Processes may arguably be the more relevant aspect of GO in relation to this study, therefore, only functional clusters belonging to this component have been presented. Comparatively induced genes from liver when individually compared to the other three tissues showed GO BP enrichments associated with cellular biosynthesis, cellular lipid metabolism, coagulation, hemostasis, metabolism, nitrogen compound metabolism, and physical process (Figure 4A). GO BP enrichment analysis of repressed liver genes for each of the three comparisons identified the categories of actin cytoskeleton organization and biogenesis, cell differentiation, cell organization and biogenesis and development. There were many significantly enriched functional categories associated with comparatively repressed genes when only considering the comparisons between liver and spleen, and liver and cecal tonsil. These were cellular physiological processes, primary metabolism, macromolecule biosynthesis, macromolecule metabolism, development, protein biosynthesis, protein metabolism, and regulation of cellular process (Figure 4B). GO BP enrichments of induced genes in the spleen revealed enriched categories that included biopolymer metabolism, nucleobase, nucleoside, nucleotide and nucleic acid metabolism, physiological process, and primary metabolism (Figures 4B, 5). Induced genes in comparisons of cecal tonsil with liver and ileum showed functional enrichment primarily categorized as cell death (Figures 4B, 5). Comparisons of repressed genes in cecal tonsils with both spleen and liver showed enrichments associated with physiological process and response to stress (Figures 4A, 5). In the functional comparisons of induced genes from ileum with both spleen and liver, there were enrichments associated with development (Figures 4B, 6); however, the repressed genes in ileum when compared to spleen and liver showed functional enrichment of cellular biosynthesis, physiological process, and protein biosynthesis (Figures 4A, 5).

### Quantitative real time PCR

To validate the microarray results, quantitative real time PCR (qRT-PCR) assays were performed on the same RNA samples used for the microarrays. A total of 23 genes were selected for these verifications. These genes included induced and repressed genes that were significantly and non-significantly expressed (Table 2). The relative signal intensities of those genes selected for qRT-PCR ranged from low to high (10 to 65535). Glyceraldehyde-3-phosphate dehydrogenase (GAPDH) was used as the normalization standard.

**Table 2:** The gene symbol, accession number, and primers of genes used for quantitative RT-PCR

Gene symbol	Accession No.(GenBank)	Forward primer (5'-3')	Reverse primer (5'-3')
IGJ	AB025103	GAAGGGAAGGCAAGATGAAG	GGGACGAACTTTGAGGTCAC
TF	AB215094	AACAACCTCAGGGACCTCAC	GTCCAAGCTAATGGCATCTG
GSTA3	AF133251	CAGAGCCATCCTCAGCTACA	CTCTGTTGCCTTCTCTGCAA
LCP2	AF226988	TGAATCACCAACGGAAGAAA	ACTGGAGGCTGATGTGATGA
CXCL14	AF285876	GTGCTTAGCCAGTGCAGAAG	TCCTCCACACAAAATGGGTA
Lb-FABP	AF380998	CTGGCAGGTCTATGCTCAAG	CACAGTCTGCCTGGGTGTT
FABP1	AF380999	TTCTCTTGTGTTGGGAGCAC	TTCCCATTCTGCACAATTTC
FIGF	AF479650	CTCGGTTCCTTTGACAAGTG	CTATCCCAGACCCATCCATT
CYP3A37	AJ250337	ATACGGACAGACCTCCAAGC	GAATGCCCAGCTTCTTGAAC
MARCO	AJ271377	CAGATGCAGAGGAGATGGAA	GCAGCAGGTAGATGACAAGG
BCMO1	AJ271386	AAACTTCCAACTTCCGCAAC	GTAGGAACTGGGCTCCACTG
TIMD4	AJ719444	GTCAGCCAAAATGTCCCACT	GTCCTTCTCTCGTGCTACCC
TNFSF10	AJ720191	TGGCCGTCACCTACATCTAC	TCAGCCACTCTGTCTTTGCT
LEAP-2	AY534899	TTCTGAGACTGAAGCGGATG	GAGGCCGTTCTAAGGAAGC
TACSTD1	BU137789	CCCTGGAGATGTGGATATAACTG	TCTGGTGGTACTTCATCAACG
HRSP12	BX932754	GCTTGTAGACCGGACCATGT	GCAGCTTTCAGGATTTCTCC
C1QA	BX934534	CATGGAACAGCTTGAGGATG	GCCCTGATTCACCTTTGTCT
GAL10	CR388516	GCTCTTCGCTGTTCTCCTCT	CCCAGAGATGGTGAAGGTG
VIP	U09350	CTGTCAAACGCCACTCTGAT	CGAAGTTTGGCTGGATTTAAC
C3	X69470	GACGTCCAGGACCTGAAGAG	CCAGGTAGATGATGAGGTTGC
VLDLR	X80207	ATGTGAGGAGTCCCAGTTCC	CCATCACACTGCCATCTGTT
CD5	Y12011	ACAGGAGGCTGATGAAGAGG	TGAGCGTAATCGTTGTCTCC
K60	Y14971	CGATGCCAGTGCATAGAGAC	GTCCAGAATTGCCTTGATGA
GAPDH	NM_204305	GAGGGTAGTGAAGGCTGCTG	CATCAAAGGTGGAGGAATGG

IGJ: Immunoglobulin J polypeptide, linker protein for immunoglobulin alpha and mu polypeptides; TF: Transferrin; GSTA3: Glutathione S-Transfer A3; LCP2: Lymphocyte cytosolic protein 2 (SH2 domain containing leukocyte protein of 76 KDA); CXCL14: Chemokine (C-X-C motif) ligand 14; Lb-FABP: Liver basic fatty acid binding protein; FABP1: Fatty acid binding protein1, liver; FIGF: 'C-fos induced growth factor (vascular endothelial growth factor D); CYP3A37: Cytochrome P 450 A 37; MARCO: Macrophage receptor with collagenous structure; BCMO1: Beta-carotene 15, 15'-monooxygenase 1; TIMD4: T-cell immunoglobulin and mucin domain containing 4; TNFSF10: Tumor necrosis factor (ligand) superfamily, member 10; LEAP-2: Liver expressed antimicrobial peptide 2; TACSTD1: Tumor associated calcium signal transducer 1; HRSP12: Heat responsive protein 12; C1QA: Complement component 1, Q subcomponent, alpha polypeptide; GAL10: Beta-defensin 10; VIP: Vasoactive intestinal peptide; C3: Complement component 3; VLDLR: Very low density lipoprotein receptor; CD5: CD5 protein; K60: K60 protein; GAPDH: Glyceraldehyde 3-phosphate dehydrogenase.

The coefficient of variation between the replicate qRT-PCR reactions was calculated and ranged from 0.1%-2%. For the genes with *P *< 5 × 10^-4 ^in microarray results, 95.5% of the genes tested by qRT-PCR were also differentially expressed (*P *< 0.05); for genes 5 × 10^-4^<*P *< 0.05 in the microarray only 16.7% were significantly and differentially expressed (*P *< 0.05) using qRT-PCR; none of the genes with *P *> 0.05 were shown to be differentially expressed using qRT-PCR. In terms of regulation direction for the genes in each qRT-PCR comparison, the microarray results were always consistent with qRT-PCR for genes with *P *< 0.0001, but only 75% when considering genes with 0.0001 <*P *< 0.05, and genes with *P *> 0.05, we found 78.57% of genes were consistently expressed between microarray and qRT-PCR (Tables 3 and 4). The fold-changes (log2 ratio) of each gene for six comparisons are presented in Tables 3 and 4. Most of fold-changes in qRT-PCR were higher than those seen in microarray comparisons.

**Table 3:** Microarray and qRT-PCR results of 23 selected genes for each pair of comparison

Gene symbol	Liver vs. spleen		Spleen vs. cecal tonsil		Cecal tonsil vs. ileum	
	
	Microarray	qRT-PCR	Microarray	qRT-PCR	Microarray	qRT-PCR
IGJ	**-4.9 **^#^	**-7.1**	2.3 ^#^	2.3	3.0 ^#^	2.2
VLDLR	**-3.2 **^#^	**-3.5**	1.6 ^#^	2.1	0.2	0.7
LEAP-2	**4.5 **^#^	**10.0**	-1.0 *	-5.5	-2.8^#^	-4.5
Lb-FABP	**8.6 **^#^	**12.6**	-0.8	0.0	-0.3	-1.0
TACSTD1	1.9 ^#^	4.1	**-5.7 **^#^	**-3.5**	0.0	-2.9
CYP3A37	3.1 ^#^	14.8	**-3.6 **^#^	**-14.8**	-0.1	0.2
C1QA	-0.5 *	-0.6	**2.0 **^#^	**2.8**	0.2	-0.2
MARCO	-0.6 *	-3.7	**2.7 **^#^	**4.7**	0.1	1.4
BCMO1	1.8 ^#^	4.8	0.8 *	-1.3	**-2.1 **^#^	**-2.6**
K60	-0.9 ^#^	-2.3	-0.8 ^#^	-1.6	**2.0 **^#^	**3.0**
GSTA3	3.1 ^#^	7.0	-3.4 ^#^	-3.9	**2.3 **^#^	**1.0**
FIGF	-1.0 *	-0.6	-2.6 ^#^	-1.8	1.0 *	0.9
CD5	-3.2 ^#^	-4.2	0.3	0.6	2.6 ^#^	2.2
GAL10	5.0 ^#^	12.7	-0.1	-2.1	0.0	-1.1
TF	4.2 ^#^	7.2	0.9 *	4.4	0.6	0.7
VIP	0.0	-2.2	-3.2 ^#^	-3.7	-0.6	-0.6
CXCL14	0.1	-5.8	-2.6 ^#^	-6.4	0.3	0.0
HRSP12	3.7 ^#^	6.0	-0.8 *	-1.8	0.3	-0.8
C3	2.8 ^#^	3.0	0.2	1.7	0.6	3.0
FABP1	8.6 ^#^	13.5	-0.8 ^#^	-6.0	-0.3	-2.7
TNFSF10	1.4 ^#^	1.3	-3.3 ^#^	-3.3	0.6	0.2
LCP2	-2.0 ^#^	-0.5	1.1 ^#^	1.0	1.1 *	-0.1
TIMD4	0.0	-0.1	2.0 ^#^	2.5	1.4 ^#^	1.0

**Table 4:** Microarray and qRT-PCR results of 23 selected genes for each pair of comparison

Gene symbol	Liver vs. cecal tonsil		Liver vs. ileum		Spleen vs. ileum	
	
	Microarray	qRT-PCR	Microarray	qRT-PCR	Microarray	qRT-PCR
IGJ	-2.6^#^	-4.9	0.4	-2.6	5.3 ^#^	4.5
VLDLR	-1.5 ^#^	-1.4	-1.4	-0.7	1.8 ^#^	2.8
LEAP-2	3.5 ^#^	4.5	0.6	0.0	-3.8 ^#^	-10.1
Lb-FABP	7.8 ^#^	12.7	7.6 ^#^	11.7	-1.0	-0.9
TACSTD1	-3.8 ^#^	-0.3	-3.7 ^#^	-3.3	-5.7 ^#^	-6.8
CYP3A37	-0.5	0.0	-0.6 *	0.2	-3.7 ^#^	-14.6
C1QA	1.5 ^#^	2.2	1.7 ^#^	2.0	2.3 ^#^	2.6
MARCO	2.1 ^#^	2.2	2.2 ^#^	3.5	2.8 ^#^	6.0
BCMO1	2.6 ^#^	3.0	0.6 *	1.0	-1.2 ^#^	-3.8
K60	-1.7 ^#^	-3.9	0.3 *	-0.9	1.2 ^#^	0.7
GSTA3	-0.2	3.0	2.0 ^#^	4.0	-1.1 *	-2.9
FIGF	**-3.6 **^#^	**-2.4**	-2.7 ^#^	-1.5	-1.6 ^#^	-0.9
CD5	**-2.9 **^#^	**-3.7**	-0.2	-1.4	2.9 ^#^	2.8
GAL10	**4.9 **^#^	**10.6**	4.9 ^#^	9.5	-0.1	-3.2
TF	**5.2 **^#^	**9.6**	5.8 ^#^	10.6	1.5 *	3.1
VIP	-3.2 ^#^	-5.8	**-3.8 **^#^	**-6.4**	-3.8 ^#^	-4.3
CXCL14	-2.5 ^#^	-11.1	**-2.3 **^#^	**-9.2**	-2.3 ^#^	-5.9
HRSP12	2.9 ^#^	3.6	**3.2 **^#^	**3.4**	-0.5	-2.6
C3	3.0 ^#^	4.7	**3.6 **^#^	**7.7**	0.8 *	4.8
FABP1	7.8	3.6	7.6	0.9	**-6.4 **^#^	**-8.1**
TNFSF10	-1.9 ^#^	-2.0	-1.4 ^#^	-1.8	**-2.7 **^#^	**-3.1**
LCP2	-0.9	0.5	0.2	0.4	**2.2 **^#^	**1.0**
TIMD4	2.0 ^#^	2.8	3.4 ^#^	3.8	**3.4 **^#^	**3.9**

Note: All data are shown as log2, positive value means up regulated between tissue A vs. tissue B (tissue A – tissue B)

^#^represents *P *value for the comparison is less than 0.0001 in microarray results.

* represents *P *value for the comparison is between 0.05 and 0.0001 in microarray results.

Micrroarray results shown in bold font in diagonal are the results those were used to select genes for qRT-PCR for each comparison.

### Utilization of the array

MIAME information about this chicken transcriptome microarray has been deposited in NCBI's Gene Expression Omnibus (GEO) [30]. The accession numbers are: Platform, GPL4993; Series, GSE7452; Samples, GSM180391–GSM180406, GSM180426, GSM180428, GSM180430, GSM180433, GSM180434, GSM180436, GSM180438, GSM180441.

## Discussion

### Microarray performance

Three different types of microarrays have been widely utilized in genome research including cDNA (long strands of amplified cDNA sequences), short oligonucleotide (25–30 nt), and long oligonucleotide (50–80 nt). Several studies have compared the performance of different platforms [10,13,31-34]. Annotation and identity of the commercial oligonucleotides are reliable and the probe performance is excellent [32]. Commercial microarrays can provide higher precision than homemade microarrays [33]. This custom long-oligo array was generated by the Agilent SurePrint ink-jet technology, which also provides a flexible platform for revising and updating oligonucleotide probes in the array without additional cost [25,35]. Only small amount of RNA is needed for labelling (50 ng to 5 μg of total RNA or 10–100 ng of poly (A)^+ ^RNA) [35], compared to at least 20–30 μg total RNA using cDNA array. This is especially important for those applications that generate limited amounts of RNA, such as laser-capture.

Chicken, as a major food animal, plays a key role in nutrition and food safety for human health, and is a model organism in developmental biology and for disease research including virology, oncology, and immunology [36]. There were several chicken whole genome microarrays as noted in the introduction. The currently described 44K long oligonucleotide (60-mer) microarray has shown overall high array quality and specificity compared to cDNA and 25-mer oligo arrays [37]. In addition, the 4 × 44K platform in the array design has the feature of four independent arrays in one slide, which is more cost effective and can also reduce variations among the arrays within a slide. The design of this array was based on expressed sequences selected by walking over the chicken genome sequences in the UCSC genome browser. This manual approach allowed us to maximize genome coverage and minimize gene redundancy.

High background levels in an array platform can obscure the signal from low-expressed genes and impede accurate quantification. The magnitude of SNR can affect the sensitivity of the microarray, and a higher SNR indicates high sensitivity and low background. In general, SNR > 3 was used as the lower-bound threshold for spot detection [21] in the current microarray studies and a SNR > 10 was the indication of high quality spots [28]. More than 95% of the spots with SNR > 10 in the array compared to 86.3 to 88.9% with SNR > 3 for the chicken cDNA array [21] have demonstrated the high sensitivity of the current array. The average SNR of the current microarray was 921.93, which was much higher than the SNR of most cDNA array platforms (35.1 to 38.3). This will promote sufficient signal generation for the detection of even low copy genes.

Quantitative real time PCR has become the gold standard for the gene expression and generally used to validate the microarray results [38]. At the criterion of *P *< 5 × 10^-4 ^in the microarray analysis, false positives could be effectively controlled (95.5% consistency between microarray and qRT-PCR). For those 4.5% inconsistent ones, large variations were observed between four biological replicates within each tissue using the more sensitive qRT-PCR method, which caused higher *P *values. On the other hand, the results from qRT-PCR demonstrated that type II errors (false negatives) can be controlled, given certain cut-off *P *value from microarray analysis (100% true false, given *P *> 0.05). These results indicated that microarray analyses from the current array were statistically reliable and accurate.

### Genes on the microarray

This whole genome 44 K microarray consists of probes designed from all potential genes and was designed based on the February 2004 chicken (*Gallus gallus*) v1.0 draft assembly. The current array design includes all of the available (150) chicken microRNAs from miRBase 8.1 [39,40], all known Marek's disease virus and two avian influenza virus (H5N2 and H5N3) transcripts. This array platform will provide a unique opportunity to study host-pathogen interaction using the same array simultaneously. This is important as we currently face potential emergence of an avian influenza virus epidemic. A second version of this array based on May 2006 chicken (*Gallus gallus*) v2.1 draft assembly has been updated and is now available.

A strict statistical criterion has been applied in the current analysis. Several thousand genes were differentially expressed between every two tissue comparison even at *P *< 10^-7^. Because there were more than 40 thousand genes analyzed in this microarray experiment; therefore, it is important to control the proportion of false positives [41]. False discovery rates (FDR) based on *P *values is the expected proportion of true null hypotheses rejected in relation to the total number of null hypotheses rejected [42]. FDR is a more convenient and natural scale than the *P*-value scale, and it can provide the probability of a gene value to be false positive [43]. In this study, the FDRs were less than 5% for a *P *value of 10^-7^, which demonstrated the reliable results of the current microarray experiment. Similar FDR were observed in gene expression profiling between different tissues using a long oligo swine array [12].

Gene expression profiles of different normal tissues provide information about the biological function of the tissue and are expected to be conserved during evolution. Liver, spleen, and ileum have been widely utilized in gene expression profiling studies in human [29,44-46] and swine [12,47]. There were some common gene ontology terms enriched with tissue comparison between spleen and ileum in both human [44] and chickens such as protein biosynthesis, energy pathways, and immune response. But there were some distinct enrich terms between human and chickens including cytochrome C oxidase activity in human, and cell death, development, M phase, macromolecule metabolism, and physiological process in chicken. For the comparison of liver and spleen, energy pathways, main pathways of carbohydrate metabolism, and fatty acid oxidation were enriched in human [44], while generation of precursor metabolites and energy, cellular carbohydrate metabolism, cellular lipid metabolism, tricarboxylic acid cycle organic acid metabolism were enriched in chickens (Figures 4A, B).

Cecal tonsil and ileum are two proximal tissues in the digestive tract. Both of these are critical components of gut-associated immune system. They might share many common functions, which means, there might be fewer genes differentially expressed between them. The lowest number of differentially expressed genes (358) was found in the comparison of cecal tonsil and ileum. These findings support the expectation that tissues with similar gene expression might have similar biological functions.

Liver is a major organ with important biological functions like lipid metabolism. More genes specifically expressed in liver than spleen and ileum and/or other tissues in human and swine [12,44,46]. Similar results were observed in chickens. In human, oxidoreductase activity, lipid metabolism, complement activation, steroid metabolism, alcohol metabolism, cytochrome P450 activity, urea cycle, coagulation, amino acid metabolism, bile acid biosynthesis, and carbohydrate metabolism were liver-selective [29,44,45,48]. In swine, coagulation pathway, alcohol metabolism, lipid processing, bile metabolism and xenobiotic metabolism were liver-specific [12]. In the current study, energy, metabolism, especial fatty acid metabolism-related genes, fatty acid or lipid binding protein, fatty acid synthase, cholesterol hydroxylase, lectin, adenyl nucleotide binding, ATPase, hydrolase, coagulation factor, cytochrome P450, lyase, C3, C4B, C8, phosphorylase, and oxidoreductase, were found liver-selective (see additional file 3).

Spleen is one of the major immune organs. Many immune-related genes were more highly expressed in spleen than the other three tissues in chickens. Similar results have been observed using northern blot hybridization [49], moreover, it was reported that immune response genes were selectively expressed in human spleen [44] and porcine small intestine [12]. Ileum is one of the more important tissues involved as part of bacterial pathogenesis studies in agricultural animals. Genes related to interaction between organisms and viral life cycle were specifically expressed in porcine ileum cDNA libraries [50]. In chickens, class II histocompatibility antigen, B-L beta chain and C7 were found ileum-selective (see additional file 2). No ileum-selective genes were available in human from the previous studies. The conserved gene expression profiles in tissue comparisons among species have provide a solid basis for comparative genomics study. The tissue-selective genes could be potentially used as markers for the origin of pathogen, like gut-related pathogens.

Perhaps one of the most important and interesting findings in the study was in relation to antimicrobial peptides (AMPs). AMPs are essential for the innate immune response in plants, flies, mammals, and chickens. There are two major families of AMPs: defensins and cathelicidins. Fourteen β-defensins, known as gallinacins (GAL) and cathelicidin have been described in chickens [51-53]. In the present study, GAL1, GAL2 and GAL6-7 showed strong comparative induction in spleen and weakest expression in the ileum. Macrophage receptor with collagenous structure (MARCO) mediates alveolar macrophages to bind, ingest and clear the inhaled particle and bacteria [54]. MARCO only expressed on the marginal zone macrophage of the spleen and macrophages of meullary cord in lymph nodes in normal mice [55]. The current study corroborates this as we also found MARCO was highly expressed in spleen compared to other tissues.

To our knowledge, this is the first study to characterize tissue expression in chickens using a whole genome array. A total of four tissues were selected for this study. Two of these (liver and spleen) are complete organs, which play significant roles in many sophisticated biological functions of the animals. The liver is responsible for lipid, amino acid, and carbohydrate metabolism, while the spleen is an essential part of immune function in animals. The other two tissues (ileum and cecal tonsil) may have less complicated functions than liver and spleen. The GO analysis of global gene expression profiling among these four tissues supported the notion that more clusters of genes would be significantly enriched in the comparisons of organ (liver and spleen) against tissues such as ileum and cecal tonsil (Figures 4A, 4B, 5). The majority of functional enrichments associated with gene regulation in the liver comparisons were consistent with the roles of liver [56]. In the spleen, there were many immune-related (cell death, apoptosis, response to stimulus etc) clusters enriched. In summary, the results above demonstrated that this newly developed chicken 44K whole genome array is a powerful genomic tool to investigate different biological processes in chickens.

## Conclusion

We have characterized a newly developed chicken 44K whole genome oligonucleotide microarray using four major tissues. This microarray in theory consists of probes designed from the whole chicken transcriptome as well as 150 microRNAs, the entire genome sequences of Marek's disease virus and two avian influenza virus genomes. Comparison of gene expression among 2 organs and 2 tissues has been submitted to GEO providing valuable comparative gene expression data to the scientific community. Novel findings related to defensins and cathelicidin expression in the spleen is highlighted. Additionally, the custom tracks for sequences and probes used in this array have been built for Chicken Genome Browser Gateway in UCSC providing an efficient tool to link genomic information from this powerful genome browser to our expression data. This array will be a complimentary platform for the scientific community to study genetics, immunology, developmental biology, genomics, nutrition, and food safety in chickens.

## Methods

### Tissue collection

Cecal tonsil (C), ileum (I), liver (L), and spleen (S) were collected from six two-week commercial broilers. Total of 24 samples were immersed into 10 volumes of RNAlater (Ambion, Austin, USA) and stored at -20°C until RNA isolation.

### Microarray design

**Figure 7:**
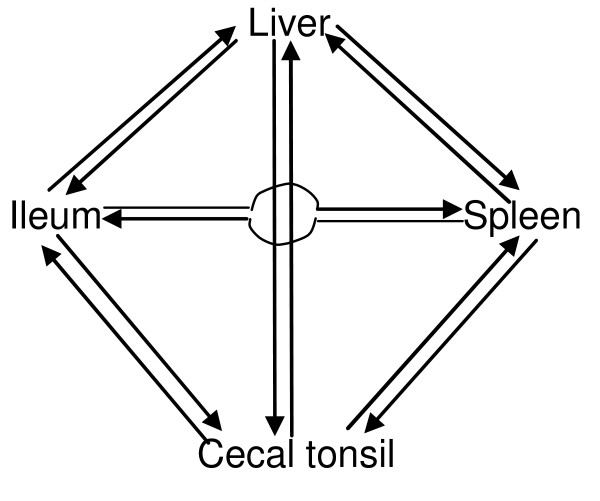
**The diagram of the microarray experiment design**. The arrow represents Cy3, the end of the arrow represents Cy5.

Loop design and dye swap were used in the microarray study (Figure 7). In brief, four different tissue samples (cecal tonsil, ileum, liver, and spleen) from each chicken were designed for one loop. The orders of the tissues in different loops were changed so that there were four comparisons with a dye swap across all six pairs of tissue comparisons. Data from 12 measurements for each tissue were collected, with total of 48 measurements from 24 arrays.

### RNA isolation

Tissues were homogenized using a Tissue Miser (Fisher Scientific, Houston, TX). Total RNA was isolated from each homogenized tissue using Trizol extraction method as described by the manufacturer (Invitrogen, Carlsbad, CA). All of DNA was removed from the samples using TURBO DNA *free*™ Kit (Ambion, Austin, TX) according to the manufacturer's protocol. The RNA quantity and purity were determined by NanoDrop ND-1000 spectrophotometer at 260/280 nm (Nano Drop Technologies, Wilmington, Delaware). The integrity of total RNA was assessed with an Agilent Bioanalyzer 2100 and RNA 6000 Nano LabChip Kit (Agilent Technologies, Palo Alto, CA). The RNA Integrity Numbers (RINs) for the samples were obtained. Only RNA samples with RIN values of 6, or higher, were used for the analysis.

### cRNA preparation

A 500 ng of aliquot of total RNA was reverse transcribed into cDNA using the Low RNA Input Fluorescent Linear Amplification Kit (Agilent Technologies). The synthesized cDNA was transcribed into cRNA and labelled with either cyanine 3 or cyanine 5-labelled nucleotide (Perkin Elmer, Wellesley, MA). Labelled cRNA was purified with RNeasy Mini columns (Qiagen, Valecia, CA). The quality of each cRNA sample was verified by total yield and specificity calculated based on NanoDrop ND-1000 spectrophotometer measurement (NanoDrop Technologies).

### Microarray hybridization

Labelled cRNAs with specificity greater than 8 were used for hybridization using the *in situ *hybridization kit plus (Agilent Technologies). Arrays were incubated at 65°C for 17 h in Agilent's microarray hybridization chambers. After hybridization, arrays were washed according to the Agilent protocol.

### Image processing

Arrays were scanned at 5-μm resolution using GenePix Personal 4100A (Molecular Devices Corporation, Sunnyvale, CA) and images were saved as TIFF format. Auto Photomultiplier tube (PMT) settings were selected and adjusted to get the ratio of the overall intensities between two channels (Cy3 and Cy5) to 0.95 to 1.05. The signal intensities of all spots on each image were quantified by Genepix pro 6.0 software (Molecular Devices Corporation, Downingtown, PA), and data were saved as .txt files for further analysis.

### Normalization and statistical analysis

The signal intensity of each probe was divided by that of negative control to filter the genes which were not expressed. The signal intensity of each gene was globally normalized using LOWESS within the R statistics package [57]. A mixed model that included the fixed effects of dye (Cy3 and Cy5), tissue, and random effect of slide and array, was used to analyze the normalized data by SAS (SAS Institute, Cary, NC). *P *value and fold changes between each comparison for each gene were calculated. One tissue was included in three comparisons, the significantly expressed genes among these three comparisons were joined together to derive the selectively expressed tissue genes. False discovery rate (FDR) (q values) were calculated by R program according to Benjamini and Hochberg's method [42].

### Bioinformatics

An unreleased version of the High Throughput Gene Ontology Functional Annotation Toolkit (HTGOFAT) was utilized [58,59] to assign updated Gene Ontology [60] numbers, Enzyme Commission [61] numbers, mappings to Kyoto Encylopedia of Genes and Genomes (KEGG) Pathways [62] and updated definitions. Additionally, differentially regulated genes were mapped to Protein Information Resource (PIR) keywords [63] and COG [64] functional annotations through the use of the Database for Annotation, Visualization and Integrated Discovery (DAVID) [65]. Statistics related to over representation of functional categories were performed using DAVID, which is based upon a Fisher Exact statistic methodology similar to that described by Al-Shahrour et al [66]. A *P *< 0.001 was considered as significant.

### Quantitative real-time PCR

Both up-regulated and down regulated genes from each comparison were selected for quantitative real time PCR (qRT-PCR). Four tissue samples from each chicken, and total of sixteen samples from 4 chickens were used. All reagents for qRT-PCR were loaded by Eppendorf ep *Motion *5070 workstation (Eppendorf, Westbury, NY). The primers were designed with Primer 3 [67]. Gene symbols and primers are listed in Table 2. A 1 ug aliquot of total RNA was used to synthesize first-strand cDNA using random hexamers and Thermoscript™ RT-PCR system (Invitrogen) in a reaction volume of 20 μL. The PCR reactions were performed in a 10 ul volume containing a 1×SYBR Green Master Mix, 50 ng cDNA, 300 nM of forward primers, 300 nM of reverse primers on an ABI Prism 7900HT sequence detection system (Applied Biosystems, Foster City, CA). The amplification condition was 50°C for 2 min; 95°C for 10 min, followed by 40 cycles of 95°C for 15 sec and 59°C for 1 min; a final soak at 4°C was also incorporated. Glyceraldehyde-3-phosphate dehydrogenase (GAPDH) was used as the internal control. All of the samples were measured in duplicate. Two measurements of each tissue sample were averaged for further analysis. The comparative Ct method was used to calculate the relative gene expression level across the tissues. Relative expression level of each gene in one tissue (ΔCt) was calculated by Ct _target gene_-Ct _GAPDH_; relative expression of each gene in two different tissues (ΔΔCt) was calculated by ΔCt _A _-ΔCt _B_.

## Authors' contributions

XL contributed to carry out the microarray experiments, analyzed data and drafted the manuscript. HC was responsible for tissue sample collection and data analysis. JZ provided the concept of the array design and contributed to the array design. SD contributed to annotation and functional analyses. HZ designed the microarray, provided the concept and design of the study, and revised the manuscript. All authors read and approved the manuscript.

## Supplementary Material

Additional file 1 **Cecal tonsil-selective expression genes**. This table includes the *P *values and fold change in each comparison for 95 selectively expressed genes in cecal tonsil. Click here for file

Additional file 2 **Ileum-selective expression genes**. This table includes the *P *values and fold change in each comparison for 71 selectively expressed genes in cecal tonsil. Click here for file

Additional file 3 **Liver-selective expression genes**. This table includes the *P *values and fold changes in each comparison for 535 selectively expressed genes in liver. Click here for file

Additional file 4 **Spleen-selective expression genes**. This table includes the *P *values and fold changes in each comparison for 108 selectively expressed genes in liver. Click here for file
